# Datasets of EQCM-controlled deposition and cycling of thin polypyrrole films in acetonitrile electrolyte solution

**DOI:** 10.1016/j.dib.2020.105360

**Published:** 2020-02-29

**Authors:** O.I. Istakova, D.V. Konev, T.O. Medvedeva, O.A. Goncharova, M.A. Vorotyntsev

**Affiliations:** a Institute for Problems of Chemical Physics, Russian Academy of Sciences, Chernogolovka, Russia; b Skolkovo Institute of Science and Technology, Moscow, Russia; c Lomonosov Moscow State University, Moscow, Russia

**Keywords:** Electropolymerization, Polypyrrole, Electrochemical quartz crystal microbalance, Conducting polymer, Polymer-modified electrodes

## Abstract

The paper presents three datasets obtained by electrochemical quartz microbalance technique which was applied to studies of conducting polymer film in contact with non-aqueous electrolyte solution. The first dataset describes the calibration procedure of gold-coated quartz crystal, immersed in acetonitrile silver ion-containing electrolyte, by means of silver layer electrodeposition. On the basis of experimentally measured dependence of the resonance frequency on the varying electrode mass in the course of electrochemical silver deposition/dissolution, the calibration coefficient was found to be equal to 13.6 ng/Hz. The second dataset has been collected when thus calibrated EQCM cell was used for determination of the mass change due to the polypyrrole film growth during anodic oxidation of pyrrole monomer from its acetonitrile solution. Its treatment reveals the proportionality between the mass change and the charge spent for pyrrole electrooxidation, the proportionality coefficient being 53.5 g per mole of electrons. The third dataset contains EQCM measurement data during repetitive charge-discharge treatment of the deposited polypyrrole film (cyclic voltammetry, CV) in monomer-free electrolyte. Collected data shows that continuous cycling of the polymer film leads to progressive increase of the cation-exchange contribution to the total ion flux which maintains the film's electroneutrality during variation of its redox state. These findings might be useful both for a qualitative consideration of the cycling stability of polypyrrole in non-aqueous medium and for a quantitative mathematical modelling of polypyrrole electropolymerization and its redox transformations.

**Table:** Specifications Table

Subject	Electrochemistry
Specific subject area	EQCM-analysis of transport processes of background electrolyte ions in conductive polymers
Type of data	Table Figure
How data were acquired	Potentiostat Autolab PGSTAT302 N (Metrohm) synchronized with QCM200 Quartz Crystal Microbalance (Stanford Research Systems)
Data format	Raw, analyzed
Parameters for data collection	Gold-coated unpolished EQCM quartz crystal (5 MHz) mounted to homemade holder. One surface of the EQCM crystal acts as working electrode in contact with an electrolyte solution inside three electrode electrochemical cell. Electrolyte solution composition: - for calibration: 0.6 mM AgNO_3_ + 0.1 М TBAPF_6_ in acetonitrile; - for electropolymerization: 0.001 M pyrrole +0.1 M TBAPF_6_ in acetonitrile; - for CV tests: 0.1 M TBAPF_6_ in acetonitrile
Description of data collection	Synchronous registration of electrochemical data (Current & Potential) and EQCM data (Analog Voltage, proportional to the resonance frequency of gold-coated quartz crystal) as functions of time. Time resolution: 10 samples per second
Data source location	Chernogolovka/Moscow region/Russia
Data accessibility	Data are included into this paper

**Table:** 

**Value of the Data** • The data provide a quantitative information on the participation of background electrolyte ions in the transport processes inside thin polypyrrole films obtained at low potentials in low-concentration monomer solutions. • The data on the in situ EQCM characterization of the processes of electrodeposition and of redox cycling of thin polypyrrole films can be useful for further understanding of the properties and patterns of ion exchange in such films both for researchers dealing with polypyrrole-coating systems and for specialists dealing with polypyrrole-based sensors. • The findings may be useful both for qualitative consideration of the cycling stability of polypyrrole in non-aqueous media and for quantitative mathematical modelling of the polypyrrole electropolymerization and its redox transformations.

## 1 Data description

### 1.1 Calibration of gold-coated quartz crystal

**Fig. 1:**
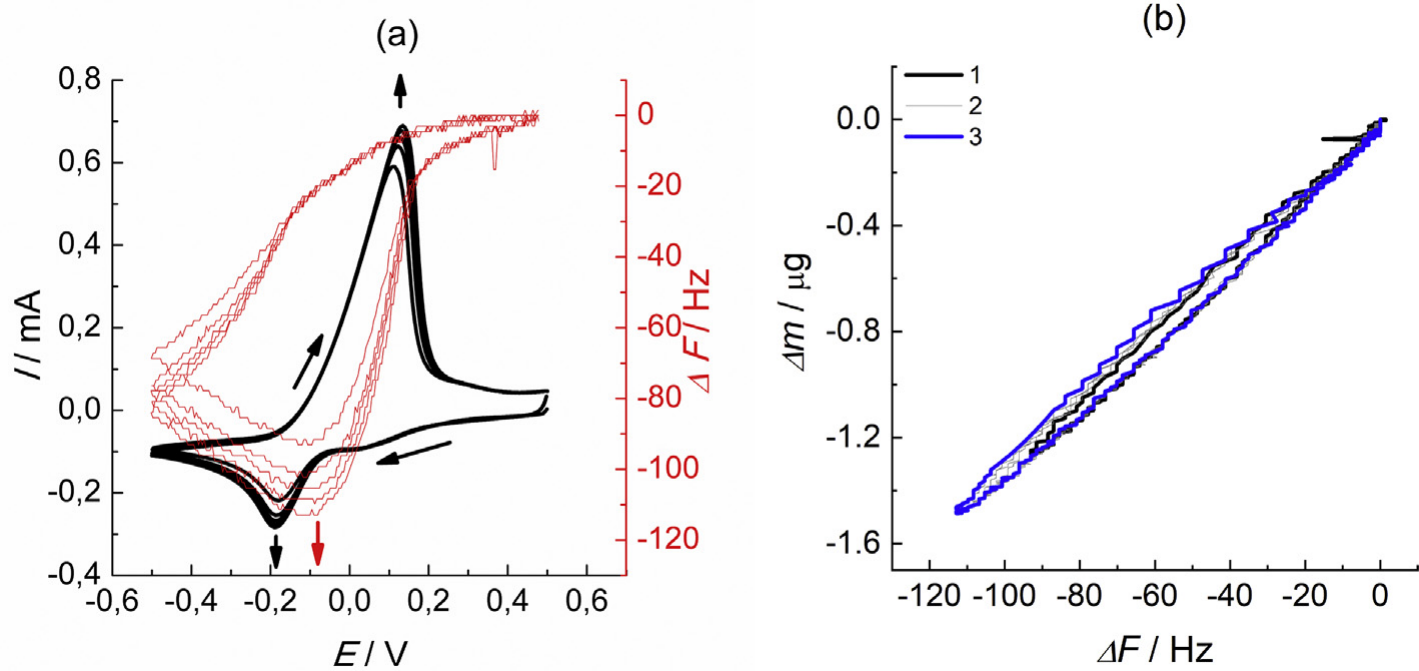
(a) Cyclic voltammogram (*I* vs. *E*, first 5 cycles, black curve) and the corresponding shift of its resonant frequency (EQCM, red curve) of gold-coated quartz crystal in contact with 0.6 mM AgNO_3_ + 0.1 M TBAPF_6_ acetonitrile solution within the potential range from −0.5 V to 0.5 V (all potentials in the paper are given vs. Ag/10 mM AgNO_3_ + 0.1 M TBAPF_6_ in acetonitrile reference electrode). Scan rate: 0.1 V/s; (b) Dependence of the mass change on the shift of the resonant frequency in the course of the cycling process: 1 – cycle 1; 2 - cycles 2–4; 3 - cycle 5. Data tables are given in attached files: Raw data for Fig.1a_dF-vs-E. Raw data for Fig.1a_I-vs-E (a). Raw data for Fig.1b_dm-vs-dF (b).

The dataset describes the calibration procedure of gold-coated quartz crystal in acetonitrile silver-containing electrolyte by means of silver layer electrodeposition. Fig. 1 presents data on the determination of the proportionality coefficient between the shift of the resonant frequency and the electrode mass change during the electroreduction of silver in the CV mode (mass change was assumed to be only due to the one-electron silver deposition, the corresponding charge being determined via integration of the CV data in Fig. 1a). This coefficient was found to be equal to 13.6 ng/Hz. This value was subsequently used for determination of the polymer film mass variation.

### 1.2 Electrodeposition of polypyrrole films

**Fig. 2:**
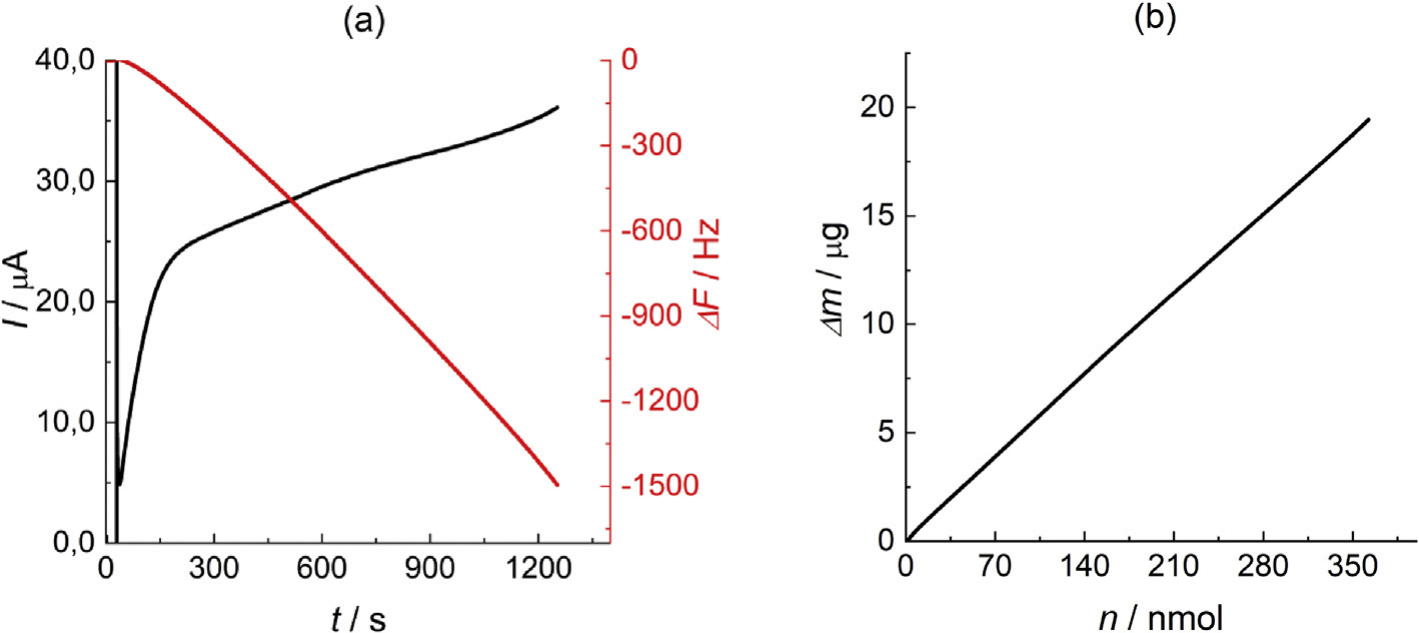
(a) Сhronoamperogram of the potentiostatic electrodeposition of polypyrrole film on the surface of the Au-coated EQCM device (black curve) and the corresponding shift of its resonant frequency (red curve). Electrodeposition was performed from 0.001 M pyrrole + 0.1 M TBAPF_6_ acetonitrile solution at the potential of 0.58 V up to the deposition charge density of 83.7 mC/cm^2^; (b) Dependence of the mass of the deposited polymer film in its charged state on the number of moles of electrons used for the monomer oxidation and for the polymer charging. Data tables are given in attached files: Raw data for Fig.2a_dF-vs-t, Raw data for Fig.2a_I-vs-t (a); Raw data for Fig.2b_dm-vs-n (b).

The dataset in Fig. 2a (chronoamperogram and shift of the resonant frequency of the quartz crystal) has been collected when the calibrated EQCM cell was used for determination of the electrode mass changes due to the polypyrrole film growth on its surface as a consequence of the anodic oxidation of the pyrrole monomer in its acetonitrile solution. Similar measurements have earlier been described by J. Heinze and coworkers [[1], [2], [3], [4]] and many scientific groups [[5], [6], [7]] for a different solution composition.

The instantaneous values of the EQCM frequency change, Δ*F*, in Fig. 2a were recalculated via the corresponding values of the deposited mass of the polymer film, Δ*m*, with the use of the above proportionality coefficient. The corresponding values of the oxidation charge (derived from the current in Fig. 2a by integration) were divided by the Faraday constant, 96500 C/mol, to determine the number of moles of electrons, *n*, passed via the circuit till this moment. Fig. 2b shows that these two quantities, Δ*m* and *n*, are proportional to one another within the whole time interval.

The slope of this dependence in Fig. 2b is 53.5 g/mol, which corresponds to the mass of the polymer film in its charged state (which includes those of the monomer units and of the charge-compensating ions) per mole of electrons. Earlier [8] in the solution of a similar composition, the redox equivalent of the pyrrole electrooxidation process was determined via the same procedure.

### 1.3 Characterization of polypyrrole film (CV test)

**Fig. 3:**
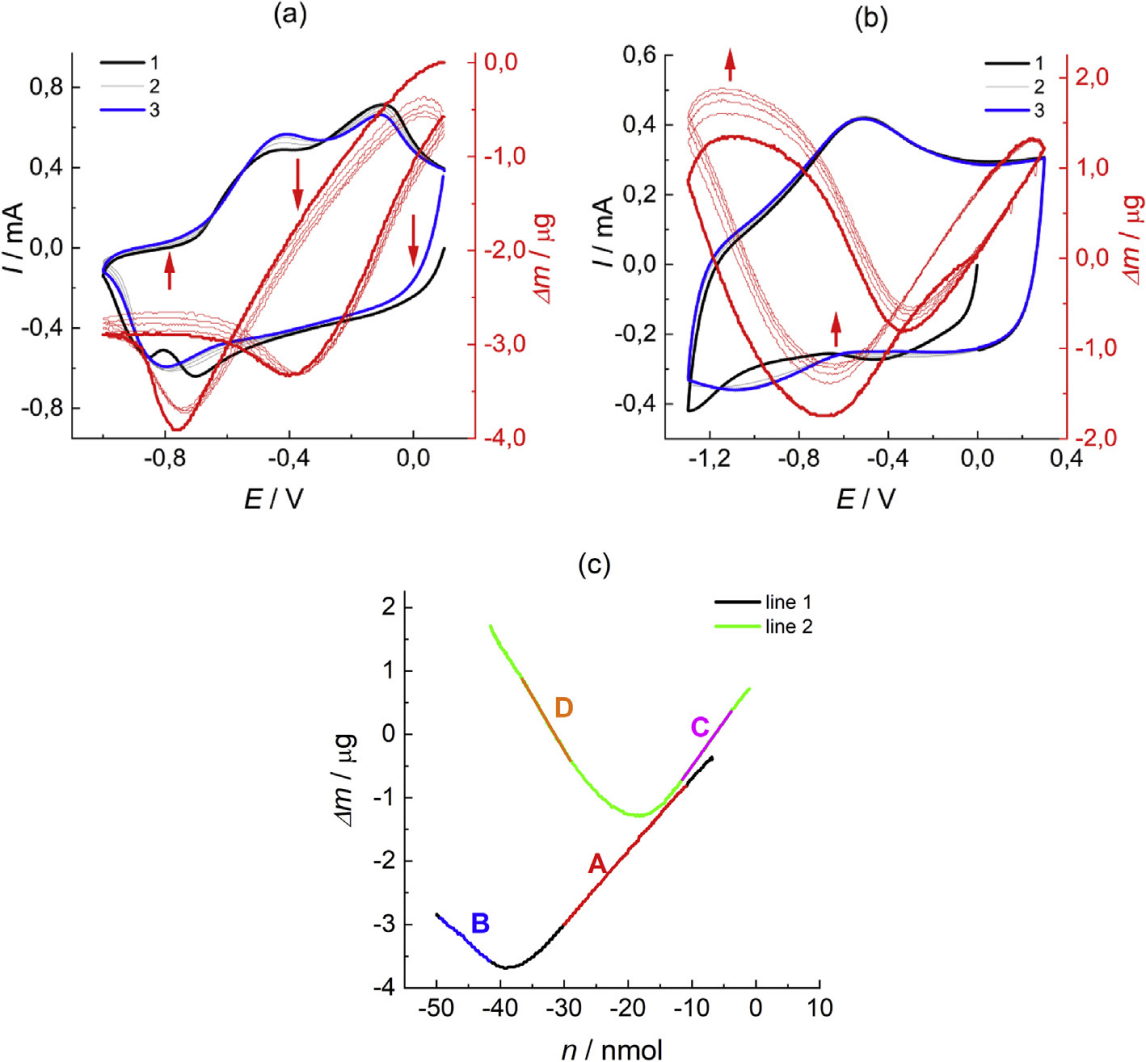
(a, b) Cyclic voltammograms and the corresponding variation of the mass change of the polypyrrole film coated quartz crystal at the beginning of cycling (a) and after 20 cycles (b) in 0.1 M TBAPF_6_ acetonitrile solution within the potential range from −1.0 to 0.1 V (a) or from −1.3 to 0.3 V (b). Scan rate: 0.1 V/s: 1 – cycle 1; 2 - cycles 2–4; 3 – cycle 5. (c) Dependence of the mass change on the number of moles of the redox charge transferred in the course of the CV procedure (data for cycle 2 in both Fig. 3a and b are used) for the polypyrrole film in contact with the monomer-free solution on the basis of data in Fig. 3a (black line 1) or in Fig. 3b (green line 2). Segments A, B, С and D show intervals of the linear variation of lines 1 and 2. Values of their slopes are indicated in Table 1. Data tables are given in attached files: Raw data for Fig.3a_dm-vs-E, Raw data for Fig.3a_I-vs-E (a); Raw data for Fig.3b_dm-vs-E, Raw data for Fig.3b_I-vs-E (b); Raw data for Fig.3c_dm-vs-n (c).

The dataset given in Fig. 3 and Table 1 contains data of EQCM measurements during repetitive charge-discharge (CV test) of the polypyrrole film (its deposition procedure has been described in section 2) in monomer-free acetonitrile electrolyte solution, with synchronous registration of the resonance frequency variation.

**Table 1:** Estimated data on the ion balance during the cycling of polypyrrole, based on Fig. 3c

Segment	Molecular weight, g/mol
segment A	115.4
segment B	87.8
segment C	140.3
segment D	170.6

Collected data in Fig. 3a, b and c show that the CV treatment (repetitive cycling of potential) of the polymer film leads to progressive increase of the cation contribution to the total ionic exchange between the film and the electrolyte solution flux which maintains the film's electroneutrality in the course of variation of its redox state. These findings might be useful both for qualitative consideration of the cycling stability of polypyrrole in non-aqueous media and for quantitative mathematical modelling of polypyrrole redox transformations.

**Fig. 4:**
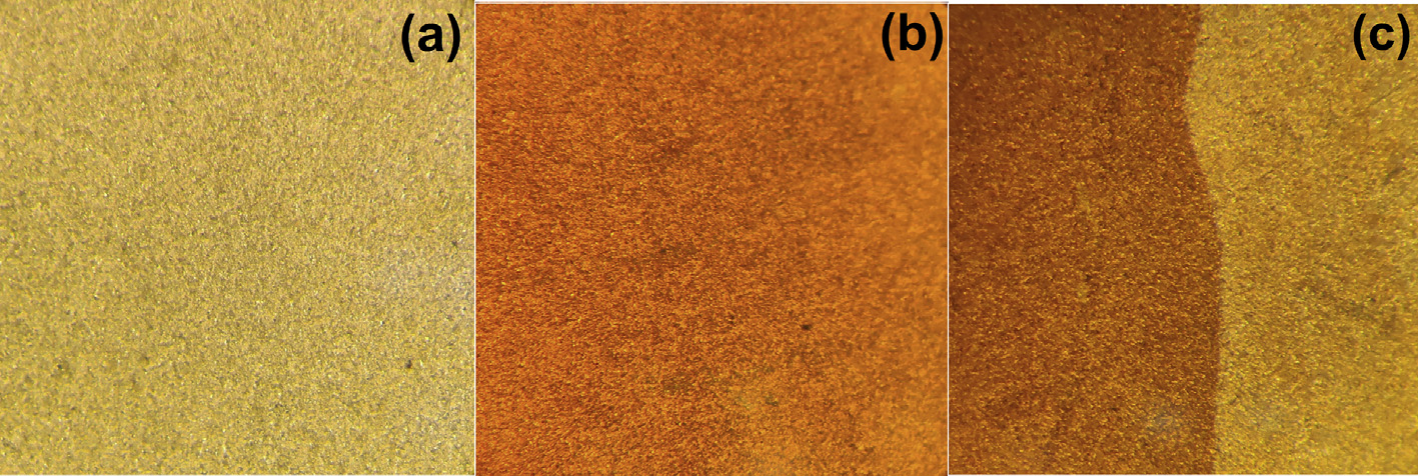
Optical microscopy images of three selected regions (each region of the 1 mm^2^ area) of the quartz crystal surface: (a) its central region coated by sputtered thin layer of gold; (b) the same region covered additionally by the polypyrrole film, see Fig. 2a for its deposition; (c) its peripherical region which was partially (left side) in contact with the solution so that it was covered by the polypyrrole film while its right side was in contact with the insulating O-ring so that no polypyrrole film was deposited there.

Optical microscopy (Fig. 4) confirms formation of a solid colored film uniformly covering the electrode surface in the range of its contact with electrolyte solution.

## 2 Experimental design, materials, and methods

Electrochemical quartz crystal microbalance (EQCM) measurements were carried out in electrochemical three-electrode cells with separated compartments, in which one of the two surfaces (covered with thin sprayed Au layers) of non-polished quartz crystal disk of 5 MHz resonant frequency was in contact with electrolyte solution and used as working electrode. The geometrical vibrationally active surface area of the working gold electrode was 0.79 cm^2^. Both electrodes on the crystal surface were connected to the SRS QCM 200 generator/analyzer of high-frequency oscillations. Potential of the working electrode immersed in the solution was simultaneously controlled by potentiostat. The two devices were synchronized in time, which made it possible to record simultaneously a chronoamperogram of the potentiostatic film electrodeposition on the working electrode (or a cyclic voltammogram) as well as the temporal variation of the resonant frequency shift of the crystal, which was proportional to the film's mass change.

All potentials are given relative to the reference electrode composed of Ag wire immersed into 10 mM AgNO_3_ + 0.1 M TBAPF_6_ acetonitrile solution. Its equilibrium potential is 100 mV more negative than that of the ferrocene (Fc/Fc^+^) redox couple in the same solution. The counter electrode was platinum wire in background acetonitrile solution separated from the working electrode compartment by porous sintered glass frit.

To calibrate the quartz crystal, AgNO_3_ (Fluka, > 99.5%) was used. Untreated acetonitrile (Panreac, HPLC) was used as solvent for preparation of calibration (AgNO_3_) and monomeric (pyrrole) solutions (sections 1, 2); it was dried with 4 Å molecular sieves prior to use in the solution for characterization of the polymer-modified electrode (section 3). The background electrolyte, TBAPF_6_ (Fluka, > 99%), was kept in oven at 80 °C for a day before the experiment. Pyrrole (Alfa Aesar, 98%) was preliminarily purified by fractional distillation under inert (Ar) atmosphere with the use of the Schlenk line.

Quartz crystal was calibrated in contact with 0.6 mM AgNO_3_ + 0.1 M TBAPF_6_ acetonitrile solution in the potentiodynamic (CV) mode within the potential range from -0.5 V to 0.5 V with scan rate of 0.1 V/s.

Potentiostatic deposition of the polypyrrole film from the 1 mM pyrrole + 0.1 M TBAPF_6_ acetonitrile solution was carried out at potential of 0.58 V. The electrolyte composition and deposition potential were selected based on papers [9,10]. The deposition charge density on the vibrationally active surface of the non-polished quartz crystal was 83.7 mC/cm^2^.

The redox response of the deposited polypyrrole films was studied by cyclic voltammetry (CV) in 0.1 M TBAPF_6_ acetonitrile solution after their thorough rinsing with pure acetonitrile to remove unreacted monomer. The redox response was recorded in the potential range from -1.0 V to 0.1 V (Fig. 3a) and from -1.3 V to 0.3 V (Fig. 3b) with scan rate of 0.1 V/s. The total number of cycles was 25. The first five cycles (Fig. 3a) and the last five cycles (Fig. 3b) are presented above. Deaeration of the solutions before the experiment was carried out with the use of the Schlenk system, by imposing vacuum briefly to the cell, followed by filling it with high purity argon.
